# Botulinum Toxin Injections for Simple Partial Motor Seizures Associated with Pain

**DOI:** 10.1155/2012/295251

**Published:** 2012-05-31

**Authors:** Edward C. Mader, Bruce J. Fisch, Nicole R. Villemarette-Pittman, Piotr W. Olejniczak, Michael E. Carey

**Affiliations:** ^1^Epilepsy Center of Excellence, Louisiana State University Health Sciences Center, New Orleans, LA 70112, USA; ^2^Department of Neurology, University of New Mexico, Albuquerque, NM 87131, USA; ^3^Department of Neurosurgery, New York University, New York, NY 10016, USA

## Abstract

Intractable epilepsy with painful partial motor seizures is a relatively rare and difficult disorder to treat. We evaluated the usefulness of botulinum toxin to reduce ictal pain. Two patients received two or four botulinum toxin (BTX) injections at one-to-two-month intervals. Patient 1 had painful seizures of the right arm and hand. Patient 2 had painful seizures involving the left foot and leg. Injections were discontinued after improved seizure control following resective surgery. Both patients received significant pain relief from the injections with analgesia lasting at least two months. Seizure severity was reduced, but seizure frequency and duration were unaffected. For these patients, BTX was effective in temporarily relieving pain associated with muscle contraction in simple partial motor seizures. Our findings do not support the hypothesis that modulation of motor end-organ feedback affects focal seizure generation. BTX is a safe and reversible treatment that should be considered as part of adjunctive therapy after failure to achieve control of painful partial motor seizures.

## 1. Introduction

Medically resistant epilepsy with frequent, painful simple partial motor seizures is a relatively rare and difficult disorder to treat. If the pain is related to extreme muscle contraction, then botulinum toxin (BTX) may be a useful treatment for pain reduction. Eisenschenk and colleagues [1] administered frontotemporal muscle BTX injections during video scalp EEG monitoring and reported a reduction in EMG artifact that allowed them to accurately localize electrographic seizure onset. Awaad and colleagues [2] found that BTX injection resulted in a dramatic reduction of painful myoclonus with functional improvement in limb movement and ambulation. Lagueny and colleagues [3] found BTX useful in treating stimulus-sensitive spinal segmental myoclonus. In contrast, Tarsy and Schachter [4] found no improvement in myoclonus or motor function in patients with epilepsia partialis continua. In this report we describe the use of BTX to control severe pain experienced by two patients during simple partial motor seizures. 

## 2. Patient 1

### 2.1. Clinical Background

A 70-year-old, right-handed male developed simple motor seizures characterized by painful dystonic posturing of the right hand following left parietal meningeoma resection. Infrequently, the motor seizure spread to the right face and leg. At baseline the patient had a right hemiparesis with clasp knife rigidity of the arm, hyperreflexia, an extensor plantar response, and distal sensory loss in the right arm and leg predominantly involving proprioception. Gait was consistent with mild right-sided spastic hemiplegia. The patient's use of his right hand was limited to maintaining a grasp.

### 2.2. Pain Associated with Seizures

 During seizures, the strength and duration of muscle contraction appeared to be proportional to pain intensity. At the onset, sustained contraction of the right forearm and hand muscles with dystonic posturing were associated with severe pain in the right hand and wrist. The patient usually attempted to relieve pain by passively dorsiflexing the right wrist and fingers with the left hand. Pain intensity was reported as 10/10 and associated with grimacing, moaning, and tearfulness. Seizure involvement of the right face and right leg was not painful.

### 2.3. Continuous Video/Scalp EEG Monitoring and Neuroimaging

Thirteen partial motor seizures were recorded during inpatient, continuous video/EEG monitoring. Most were not associated with epileptiform activity and either showed little change or irregular left central theta waves. Rarely, rhythmic low amplitude sharp waves appeared in the left central head region after the onset of arm jerking. MRI scanning indicated volume loss and increased T2 signal in the left parietal area.

### 2.4. Treatment: Vagus Nerve Stimulation (VNS) and Botox Injection

Initially, VNS plus levetiracetam resulted in a 70% reduction in seizure frequency. The VNS was turned off to allow MRI scanning, and its initial effectiveness was never reestablished. The patient received four BTX injections spaced 1 to 2 months apart using the following dosing schedule: (1) 200 units, right forearm, (2) 50 units, right deltoid; 50 units, right biceps brachii, (3) 100 units, right forearm flexor muscles, and (4) 50 units, right deltoid; 50 units, right biceps brachii; 50 units right forearm flexors; 50 units, right forearm extensors. The patient reported satisfactory pain relief during seizures and ictal facial grimacing and tearfulness resolved. Epilepsy surgery was then performed and resulted in seizure control so that further injections were not necessary.

### 2.5. Results

The patient reported less-painful seizures several hours after the first injection, suggesting an early onset of toxin-induced analgesia or placebo effect. However, he reported the same degree of sustained relief (80–90%) prior to injections two, three, and four as he did at one week after first injection. Thus, pain did not return to baseline levels between injections, so duration of effect following each injection was at least two months. Pain during seizures was easily tolerated by the patient but continued to vary directly with seizure duration and intensity. Definitive resective surgery occurred one month after the fourth injection.

## 3. Patient 2

### 3.1. Clinical Background

 A 30-year-old male presented with a 27-year history of partial motor seizures with painful muscle contractions beginning in the left foot and leg, sometimes spreading to the left thigh. The contractions occasionally extended rostrally to the thoracic paraspinal muscles. He also experienced complex partial, secondarily generalized seizures and simple partial status epilepticus. His neurological exam was normal.

*Pain Associated with Seizures*. The patient reported a pain level of 10/10 during contractions of the left leg and foot at seizure onset. Muscle contractions in the left thigh, as well as in the paraspinal region, elicited a similar pain rating.

### 3.2. Continuous Video/Scalp EEG Monitoring and Neuroimaging

Inpatient, continuous video/EEG monitoring recorded 10–50 partial motor seizures per day and an episode of simple partial focal motor status. Electrographic seizure onset appeared as rhythmic fast activity, C4 greater than Cz, and 2–5 seconds after clinical seizure onset. The MRI did not reveal structural abnormalities. Ictal SPECT did not show perfusion abnormalities. Interictal PET revealed hypometabolism in the medial right posterior frontal and anterior parietal lobes.

### 3.3. Treatment: Vagus Nerve Stimulation (VNS) and Botox Injection

While under our care, VNS plus phenytoin, oxcarbazepine, clorazepate, and levetiracetam failed to control seizures. The patient received two BTX injections one month apart: (1) 40 units, left gastrocnemius; 30 units, left tibialis posterior; 30 units, left peroneus longus; 20 units, left peroneus brevis; 20 units, left flexor digitorum brevis; 40 units, left paraspinal muscles; 30 units, right paraspinal muscles, and (2) 300 units to the left thigh muscles, including the vastus medialis, vastus lateralis, rectus femoris, and thigh adductors. Injections were discontinued following successful palliative epilepsy surgery. Surgery included subpial transections of the superior and medial aspects of the right cerebral hemisphere and cortical resection of the medial aspect of the right cerebral hemisphere. Because some seizures persisted, the patient was subsequently enrolled by us (B. J. Fisch and M. E. Carey) in a Phase II clinical trial for the treatment of medically refractory partial seizures.

### 3.4. Results

The patient reported a 60–70% reduction in pain in his left lower leg and paraspinal muscles during seizures two to three days after his first injection. The focus of maximum pain shifted to the left foot and thigh but the highest ictal pain rating was 4/10. The second injection included left thigh muscles, and further pain relief was reported. Toxin-induced analgesia in the areas targeted by the first injection was sustained for at least two months. Epilepsy surgery occurred one month after the second injection.

## 4. Discussion

Our patients experienced substantial relief from painful simple partial motor seizures following BTX injection. They also reported persistent pain in the untreated muscles after the first injection and requested injections in those muscle groups during subsequent treatments. When BTX was injected into these previously untargeted locations, there was further alleviation of pain. Neither patient missed a BTX appointment and BTX therapy ceased only because Patient 1 underwent successful epilepsy surgery and Patient 2 had an epilepsy surgery resulting in improvement in seizure frequency and intensity and was then enrolled in a clinical trial.

The frequency of seizures was not improved following BTX injection in either patient. Our findings are therefore in agreement with those of Tarsy and Schachter [4] who reported BTX treatment to be ineffective for reducing seizure frequency in epilepsia partialis continua. Consequently, even though BTX made the seizures more tolerable for our patients, both remained enthusiastic about trying other means of seizure control, including epilepsy surgery. Although biofeedback has been considered as a possible treatment for some forms of epilepsy [5], in this specific instance we found no evidence that the modulation of the motor end organ affects ictogenesis.

Most patients with painful muscle disorders experience some relief after treatment with BTX [6]. The mechanism of induced pain relief was initially attributed to muscle paralysis; however, there are reports in which BTX-associated pain reduction is out of proportion to the degree of reduction in muscle relaxation, as well as reports of relief in areas where there was no reduction in muscle tension [7]. These clinical observations raise the possibility that BTX-induced analgesia may involve mechanisms that are not limited to the toxin's paralyzing effect on skeletal muscle. It has been shown that BTX undergoes retrograde transport to the dorsal root and spinal cord [8], suggesting that BTX may be involved in pain modulation at the spinal cord level. BTX has also been shown to inhibit the release of substance P from trigeminal nerve endings, block glutamate release, and interfere with Fos expression in the periphery, in addition to its well-known effect of inhibiting acetylcholine release [9, 10]. These findings may help explain the rapid pain response to BTX that occurred in our patients.

Pain is not a common manifestation of seizures. True ictal pain in the absence of muscle contraction has been documented [11], and one of us B. J. Fisch has treated a patient with laboratory documented, simple partial seizures consisting of an extremely painful, unilateral burning foot sensation. Pain related to muscle contraction during seizure is more common, albeit rare. Overall seizure control remains the primary goal for treatment of painful motor seizures, as it is for any type of seizure disorder. However, when seizures are refractory to aggressive traditional treatment, additional symptomatic relief should be explored.

BTX is a safe option for symptomatic relief and for pain in particular [12], and, most importantly, its effects are typically reversible (e.g., [13]). Eisenschenk and colleagues reported no adverse effects in their account of 3 patients with BTX-A injections into the frontotemporal muscles prior to inpatient monitoring to reduce muscle artifact and improve seizure localization using EEG. Full recovery of muscle strength occurred in an average of 11.3 weeks [1]. Other studies targeting larger muscle groups have also reported few or no adverse effects. For example, in a study of 39 adolescents and adults, BTX injections were administered to the upper and lower extremities to treat acquired spasticity. The authors report no adverse effects throughout treatment [14]. In a randomized, double-blinded, controlled comparison of BTX and Neuronox for spastic equines gait with cerebral palsy, Kim and colleagues injected the calf muscles of children (BTX, *n* = 59; Neuronox, *n* = 60) and followed them for 24 weeks. Frequency of adverse effects did not differ between the treatment groups, and there was only one participant receiving BTX that experienced transient subjective muscle weakness [15]. Finally, a meta-analysis of randomized, placebocontrolled, double-blind trials of BTX in poststroke spasticity showed that there was no difference in the odds ratio of having an adverse effect between placebo and BTX treatment groups [16].

Our limited experience using BTX to reduce pain related to muscle contraction during seizure suggests that BTX may be an effective treatment. It remains unknown whether BTX injections would have any effect on localized ictal pain unassociated with muscle contraction. Future, prospective controlled studies of BTX for painful motor seizures are required to determine the true clinical utility of this palliative intervention. The safety and reversibility of BTX, coupled with its documented effects of alleviating pain, make it a feasible option for adjunctive, palliative treatment of painful motor seizures.
